# ‘Show me the money’: An analysis of US global health funding from 1995 to 2019

**DOI:** 10.7189/jogh.14.04173

**Published:** 2024-10-25

**Authors:** Madeleine Carroll, Nensi Ruzgar, Maíra Fedatto, Kurt Schultz, Maija Cheung

**Affiliations:** 1 Yale New Haven Hospital, Department of Surgery, New Haven, Connecticut, USA; 2 Beth Israel Deaconess Medical Center, Department of Surgery, Boston, Massachusetts, USA; 3 Kids Operating Room, Edinburgh/London, UK

## Abstract

**Background:**

Historically, the US has been the largest contributor to development assistance for health (DAH), although its allocation has shifted in response to outside forces. This included, for example, the establishment of the Millennium Development Goals (MDGs) in 2000, which emphasised child mortality, maternal health, HIV/AIDS, and malaria. This led to funds being earmarked for disease-specific interventions rather than health system strengthening (HSS). In 2007, the World Health Organization (WHO) published six health system building blocks, representing essential components of strong health systems. In 2015, the MDGs were replaced by the Sustainable Development Goals (SDGs), which emphasised capacity-building as opposed to specific health problems. The Lancet Commission on Global Surgery, meanwhile, highlighted surgical capacity building as essential to achieving Universal Health Coverage (UHC). Given the renewed emphasis on a comprehensive approach rather than disease-specific interventions, one might anticipate the US aligning with this rhetoric in its allocation of DAH. However, we hypothesise that this is not the case.

**Methods:**

We queried the Organization for Economic Co-operation and Development (OECD) database for allocation of US DAH to low- and middle-income countries between 1995 and 2019, thereby excluding data after 2019 to avoid the influence of the coronavirus disease 2019 pandemic. OECD entries were assigned to health systems strengthening (HSS) or disease-specific interventions categories. The WHO building blocks were used as a framework for health systems strengthening.

**Results:**

From 1995 to 1999, US DAH allocated to HSS decreased from 42% to 34%. The allocation decreased further from 34% in 2000 to 4% in 2007; correspondingly, DAH allocated to disease-specific interventions increased from 67% to 96%. Between 2008 and 2019, the distribution of US DAH remained relatively stable, with funds allocated to HSS versus disease-specific interventions ranging from 3–12% and 88–98% respectively.

**Conclusions:**

While total US DAH contributions in the 1990s and early 2000s were significantly lower compared to the decade that followed, the distribution of these funds was more evenly divided between HSS and disease-specific interventions. Despite attempts by the WHO and United Nations to redirect attention to HSS as the path to achieving UHC, the US continues to largely support disease-specific interventions and overlook the importance of HSS, including surgical capacity building.

Historically, the US has played a key role in providing financial and technical assistance to support health initiatives and programmes around the world. As the largest contributor to development assistance for health (DAH), it has channelled substantial funding and resources to improve and maintain global health. Over the last several decades, the US allocation of DAH has shifted in response to several forces and events, including humanitarian crises, global economic and political shifts, policy and budgetary changes, international commitments, and public health emergencies. In response, the pendulum of global health funding prioritisation has swung back and forth between health systems strengthening and disease-specific interventions, with the former representing a holistic growth approach to improvements in health care delivery, and the latter representing projects and initiatives targeted to specific diseases that are most prevalent or relevant to the funding sources (**Figure 1**).

**Figure 1:**
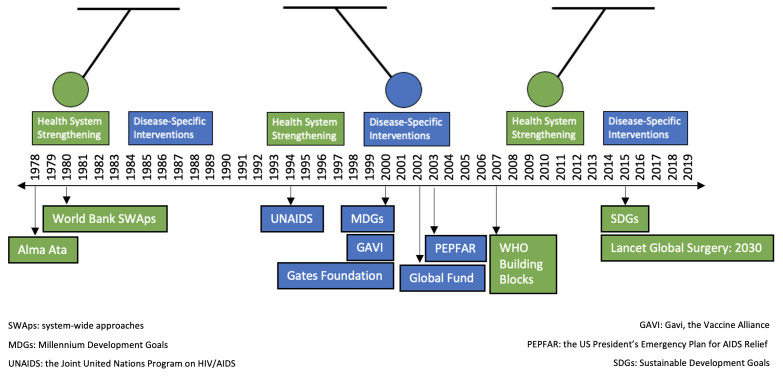
The pendulum of global health funding prioritisation (1978–2019). The green colour represents alignment with a health systems strengthening approach, while the blue colour represents alignment with a disease-specific intervention approach.

Approved and adopted in 1978, the Alma Ata declaration emphasised the importance of primary health care as the key to achieving the goal of ‘Health for All’ by the year 2000, highlighting the need for comprehensive and integrated health services that are universally accessible [1]. The declaration called for a focus on equity in health and for a more comprehensive approach to care. The declaration’s vision of achieving universal access to basic and essential health care set an ambitious goal for the global health community. While the deadline was not met, the principles outlined in the Alma-Ata Declaration have had a lasting influence on the global health agenda.

In 1980, the World Bank proposed Sector-Wide Approaches (SWAps) as a response to the widespread dissatisfaction with the fragmented nature of donor-sponsored projects. This proposal aimed to improve the efficiency and effectiveness of development assistance, emphasising ‘local ownership and partnership between the government and donors, and the holistic development of the health sector rather than numerous, independent projects’ [1]. Moreover, these SWAps encouraged donor funding to be closely aligned with countries’ national health strategies and plans as this approach would build resilient and sustainable health systems.

Beginning in the mid-1990s, there was a transition from horizontal to vertical approaches within the health sector. A major catalyst of this shift was the HIV/AIDS epidemic. This unprecedented global public health crisis led to a global response through the mobilisation of significant financial resources from various sources, including international donors, governments, and non-governmental organisations. In 1994, the Joint United Nations Program on HIV/AIDS (UNAIDS) was established, while at the UN Millennium Summit in 2000, the United Nations General Assembly established the Millennium Development Goals (MDGs) which identified the specific global health challenges of HIV/AIDS, child mortality, maternal health, and malaria [2]. Subsequently, the early 2000s saw the emergence of Gavi, the Vaccine Alliance (2000), the Bill and Melinda Gates Foundation (2000), the Global Fund (2002), and the US President’s Emergency Plan for AIDS Relief (PEPFAR) (2003) [1]. The emergence of these major funders led to a massive increase in money flowing into low- and middle-income countries (LMICs), yet this funding was earmarked for specific health issues, with HIV/AIDS being the dominant funding target. As Travis et al. [3] explained, ‘the drive to produce results for the MDGs led many stakeholders to focus on their disease priority first, with an implicit assumption that through the implementation of specific interventions the system [would] be strengthened more generally’. Unfortunately, this assumption proved false, and the over-concentration of resources in specific areas of the health sector may have exacerbated the fragility of health systems [3,4]. It became clear that channelling resources disproportionately to specific health issues often results in unintended consequences, diverting attention and funding away from other critical components of the health system, undermining their overall resilience and effectiveness and hindering their ability to address a comprehensive range of health challenges.

Following the notable swing toward disease-specific interventions in the early 2000s, the pendulum of global health discourse began to gravitate back toward health systems strengthening. In 2007, the World Health Organization (WHO) published a framework for action that consisted of six building blocks representing essential components of strong health systems, identifying health systems strengthening as a priority for global health workers and donors [1,5]. In 2009, acknowledgement by stakeholders that the MDGs would not be universally achievable without stronger health systems came in the form of the initiation of the Health Systems Funding Platform, which brought together Gavi, the World Bank, and the Global Fund to increase coordination of international resources for national health programmes [1].

In 2015, the MDGs were succeeded by the Sustainable Development Goals (SDGs). Unlike the MDGs, which target specific health issues, the SDGs emphasised the importance of capacity-building through infrastructure, economic growth, and strong institutions [6]. In the same year, the Lancet Commission on Global Surgery published its Global Surgery 2030 report, which aligned core surgical indicators to scale up surgical care with the six building blocks of robust and resilient health systems set forth by the WHO [7]. This report emerged in response to ongoing neglect of surgical diseases and non-communicable diseases (NCDs) overall in the allocation of DAH, despite countless studies demonstrating the high and increasing prevalence of these disease classes. Notwithstanding the increasing burden of NCDs, which have become the leading cause of death worldwide, the allocation of DAH has historically been skewed towards infectious diseases, maternal and child health, and certain vertical programmes.

While the prevalence and morbidity of NCDs have been increasing, the DAH allocation has not mirrored these trends. Here, we characterise the DAH contributions made by the US as the largest contributor to DAH between 1995 and 2019, and compare the changes to those observed in the disease burden. Despite calls to align DAH contributions with changing disease trends, in order to maximise the distribution of resources and provide a more comprehensive response, we hypothesise that US contributions to DAH have lagged in mirroring disease the burden or support of systems-based holistic support.

## METHODS

### DAH

**Figure 2:**
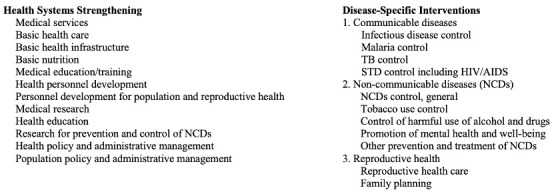
Categorisation of OECD database health sector categories. A comprehensive list of the OECD database funding allocation categories that were aggregated to calculate total funds directed to health systems strengthening vs disease-specific interventions.

To assess the allocation of US DAH to LMICs, we queried the Credit Reporting System database of the Organization for Economic Cooperation and Development’s (OECD) Development Assistance Committee for contributions between 1995 and 2019. We purposefully excluded data after 2019 to avoid the influence of the COVID-19 pandemic, as resources were expectedly shifted toward infection prevention, infection treatment, and further communicable disease efforts. OECD database categories within the health sector were grouped into the two main categories: health systems strengthening or disease-specific interventions. The determination of entries assigned to health systems strengthening was based upon the WHO building blocks framework (**Figure 2**).

### Disease burden

To assess disease burden, we obtained data from the Institute for Health Metrics and Evaluation (IHME) Global Burden of Disease 2019 report [8]. We looked at causes of death in low income and lower-middle income countries in each year from 1990 to 2019. The three broad categories of disease available in the report were communicable, maternal, neonatal and nutritional diseases; non-communicable diseases; and injuries. As our analysis aimed to compare communicable and non-communicable diseases, we considered communicable diseases as their own category and combined maternal, neonatal and nutritional diseases (individual categories which are, by definition, non-communicable) with the IHME non-communicable disease category to create a broader non-communicable disease category.

### Statistical analysis

We presented variables as absolute values and percentages and expressed them as medians (MDs) and interquartile ranges (IQRs). Yearly data was treated as a time series, and the Mann-Kendall trend test was used to analyse data over time. All statistical analyses were done in Stata version 18.0 (College Station, Texas, USA).

## RESULTS

### Total US-based DAH

**Figure 3:**
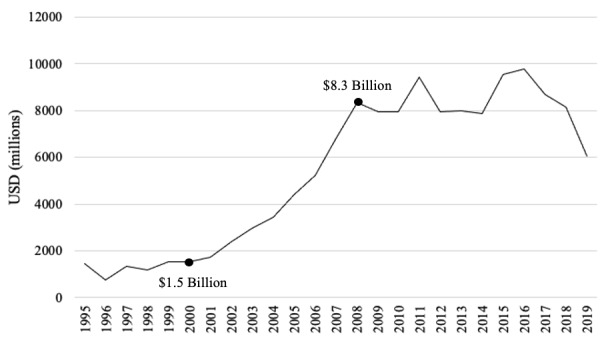
Total annual US DAH (1995–2019). A line graph showing the trend in total annual US DAH from 1995-2019 and highlighting the dramatic increase between the years 2000 and 2008.

Over the 24-year period, total annual US-based DAH rose from USD 1.5 billion to USD 6.1 billion (**Figure 3**). Notably, US DAH increased significantly between 2000 and 2007, from USD 1.5 to USD 8.3 billion. After that, funding stayed consistent, peaking in 2011 and 2014–2015 before beginning to decline, until reaching USD 6.1 billion in 2019.

### US DAH allocated to health systems strengthening vs disease-specific interventions

**Figure 4:**
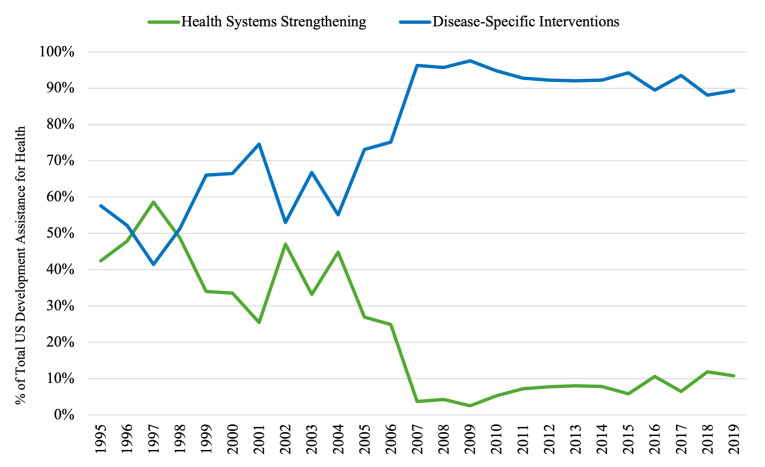
US funding of health system strengthening vs disease-specific interventions (1995–2019). A comparison of US DAH allocations to health systems strengthening (green) vs disease-specific interventions (blue) with an underlying timeline of important events in global health funding and discourse.

The percentage of US DAH allocated to health systems strengthening decreased from 42% to 34% from 1995 to 1999, with a median percentage of 47.9% (IQR = 42.4–48.8%) over this period. The percentage of DAH allocated to disease-specific interventions, meanwhile, increased from 58% to 66%, with a median percentage of 52.1% (IQR = 51.2–57.6%) (**Figure 4**). The DAH for health systems strengthening (59%) exceeded that for disease-specific interventions (41%) only in the year 1997. From 2000 to 2007, the percentage of US DAH allocated to health systems strengthening decreased from 34% to 4%, with a median percentage of 30.0% (IQR = 25.2–39.2%), with a substantial drop between 2006 and 2007 (25% to 4%). Correspondingly, the percentage of DAH allocated from 2000 to 2007 to disease-specific interventions increased from 67% to 96%, with a median percentage of 70.0% (IQR = 60.8–74.8%) over this period. The distribution of US DAH then remained relatively stable from 2008 to 2019, with funds allocated to health systems strengthening vs disease-specific interventions ranging from 3% to 12% (MD = 7.5%, IQR = 5.5–9.3%) and from 88% to 98% (MD = 92.5%, IQR = 90.7–94.5%), respectively. Over the entire period of 1995–2019, there was a significant increase in the percentage of US DAH allocated to disease-specific interventions (MD = 88.1%, IQR = 66.0–92.8%; *P* = 0.001) and a significant decrease in the percentage of US DAH allocated to health systems strengthening (MD = 11.9%, IQR = 7.2–34.0%; *P* = 0.001).

### US DAH allocated to communicable diseases vs NCDs

**Figure 5:**
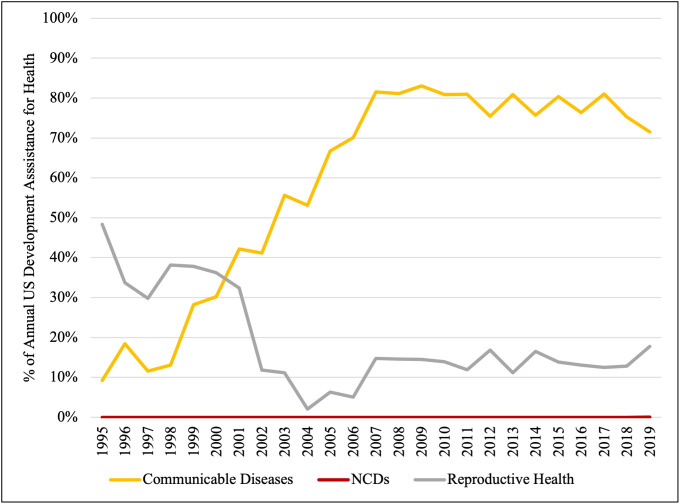
Trends in allocation of US funding for disease-specific interventions (1995–2019). A comparison of US DAH allocations to the three defined categories of disease-specific interventions: communicable diseases (yellow), NCDs (red), and reproductive health (grey).

Examining the period from 1995 to 2007, we see that the percentage of US DAH allocated to communicable diseases increased from 9% to 82% (**Figure 5**). As total US-based funding was simultaneously increasing, this meant an increase in communicable disease-directed funding from USD 135 million to USD 5.6 billion, a 41-fold increase. The percentage of US DAH allocated to reproductive health decreased from 48% in 1995 to just 2% in 2004, and subsequently increased and plateaued, never surpassing 20% through 2019. Over the entire period of 1995–2019, there was a significant increase in the percentage of US funding for disease-specific interventions allocated to communicable diseases (MD = 71.5%, IQR = 41.2–80.9%; *P* < 0.001) and a significant decrease in the percentage of US funding for disease-specific interventions allocated to reproductive health (MD = 14.5%, IQR = 11.9–29.8%; *P* = 0.034). Within the examined time period, only USD 50 000 was allocated to NCDs in a single year (2019), representing 0.0001% of total US funding in that year. However, non-communicable diseases were not included as a category in the OECD database prior to 2018, so we assume that no donors were specifically earmarking funds for NCDs before then.

### Disease burden of communicable diseases vs NCDs

**Figure 6:**
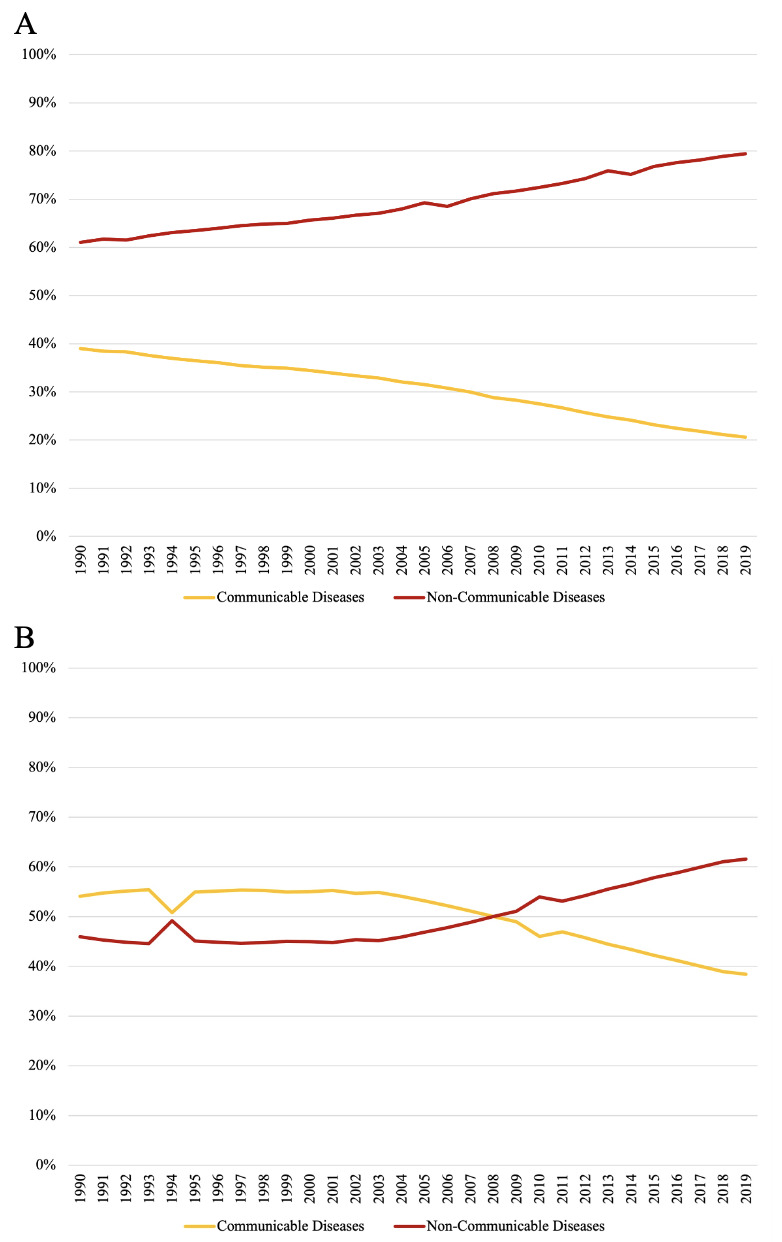
Deaths attributable to communicable vs non-communicable diseases (1990–2019). **Panel A.** Lower-middle income countries. **Panel B.** Low-income countries. A comparison of percentages of annual deaths in lower-middle income countries attributable to communicable diseases (yellow) vs NCDs (red).

According to the Lancet’s Global Burden of Disease 2019 report, the percentage of deaths in lower-middle income countries attributable to NCDs (MD =  68.2%, IQR = 64.5–74.3%) has exceeded that attributable to communicable diseases since at least 1990 (MD = 31.8%, 25.7–35.5%) and increased from 61% in 1990 to 79% in 2019 (**Figure 6**, Panel A). In low-income countries, communicable diseases caused a greater percentage of deaths (MD = 54.9%, 53.2–55.2%) than NCDs (MD= 45.2%, IQR = 44.8–46.8%) until 2008. Since then, the percentage of deaths in low-income countries attributable to NCDs has significantly risen from 51% in 2009 to 62% in 2019 (MD = 56.6%, IQR = 54.0–59.9%; *P* < 0.001), whereas the percentage of deaths attributable to communicable diseases has significantly fallen (MD = 43.4%, IQR = 40.1–46.0%); *P* < 0.001) (**Figure 6**, Panel B).

### Breakdown of communicable disease funding

**Figure 7:**
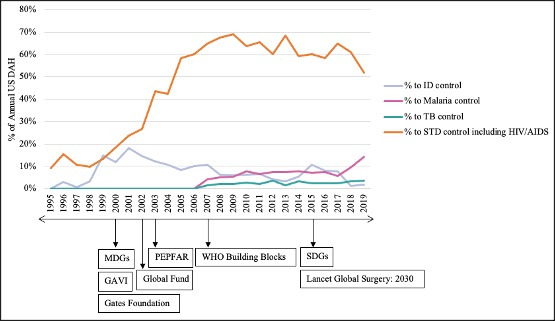
Patterns in US funding for communicable diseases (1995–2019). A comparison of the percentage of annual US DAH allocated to the four specified categories within the overall ‘communicable diseases’ category: infectious disease (ID) control (blue/purple), malaria control (pink), tuberculosis control (green/blue), and sexually-transmitted disease control including HIV/AIDS (orange). Underlying timeline of significant events in global health funding and discourse included.

From 1995 to 2007, the percentage of total US DAH allocated to ‘STD control, including HIV/AIDS’ significantly increased from 10% to 65% (MD = 23.9%, IQR = 13.4–43.5%); *P* < 0.001) (**Figure 7**). From 2008 to 2019, the percentage allocated to STD control including HIV/AIDs did decrease (*P* = 0.034), but remained above 50% in every year, ranging from 52% to 69% of total annual US DAH (MD = 62.4%, IQR = 59.7–66.6%)). There is minimal to no available data on the allocation of funds to non-HIV STDs, but based on the reviewed literature regarding HIV/AIDS funding, we presumed that most of the funds in this category were specifically allocated to HIV/AIDS treatment/prevention.

### Drivers of US DAH Allocation

**Figure 8:**
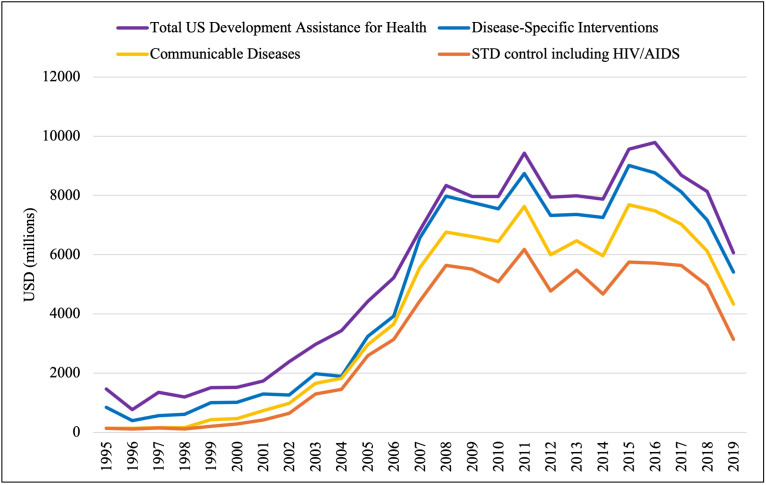
Drivers of US funding allocation (1995–2019). A comparison of the trends in total US DAH (purple), funding allocated to disease-specific interventions (blue), funding allocated to communicable diseases (yellow) and funding allocated to sexually-transmitted disease control including HIV/AIDS (orange).

We compared the trend of total US DAH allocation (MD = USD 6061 million, IQR = 1736–7991) with the trends in funding allocated to the disease-specific interventions category (MD = USD 5410 million, IQR = 1266–7549), the communicable diseases subcategory of disease-specific interventions (MD = USD 4332 million, IQR = 732–6465), and the STD control including HIV/AIDS subcategory of communicable diseases (MD = USD 3146 million, IQR = 415–5481). In visually expecting the graphs (**Figure 8**), we see that the shape of each of these curves is remarkably similar, suggesting that funding allocated to HIV/AIDS control was the main driver of the significant increase (*P* < 0.001) in total US DAH allocation from 1995 to 2019.

**Figure 9:**
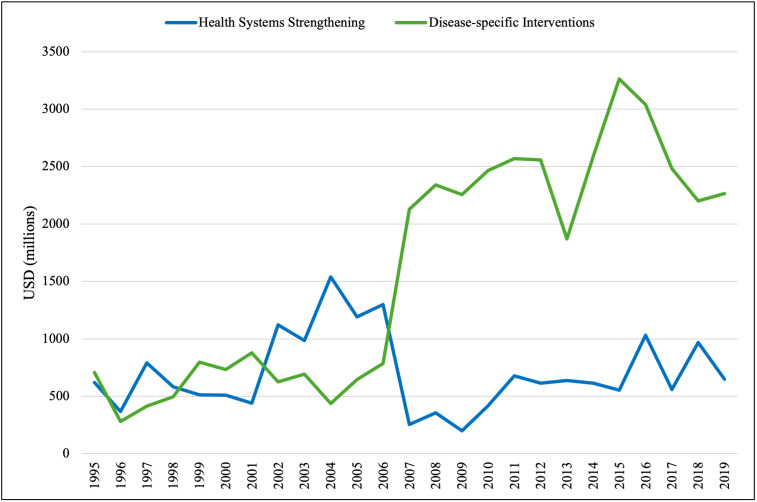
US funding of health systems strengthening vs disease-specific interventions, excluding HIV/AIDS (1995–2019). A comparison of the US DAH allocated to health systems strengthening (green) vs disease-specific interventions (blue) after removal of the HIV/AIDS subcategory from the communicable disease and in turn disease-specific intervention category.

Given this finding, we re-examined the allocation patterns of US DAH to health system strengthening vs disease-specific interventions, excluding HIV/AIDS. The previously noted pattern of a sharp and significant increase in funding directed to disease-specific interventions (MD = USD 1872 million, IQR = 692–2466; *P* < 0.001) remained, while there was no significant change in funding directed to health system strengthening from 1995-2019 (MD = USD 615 million, IQR = 511–968; *P* = 0.691) (**Figure 9**).

### NCD Funding: Comparing the US to other G7 Donor Countries

**Figure 10:**
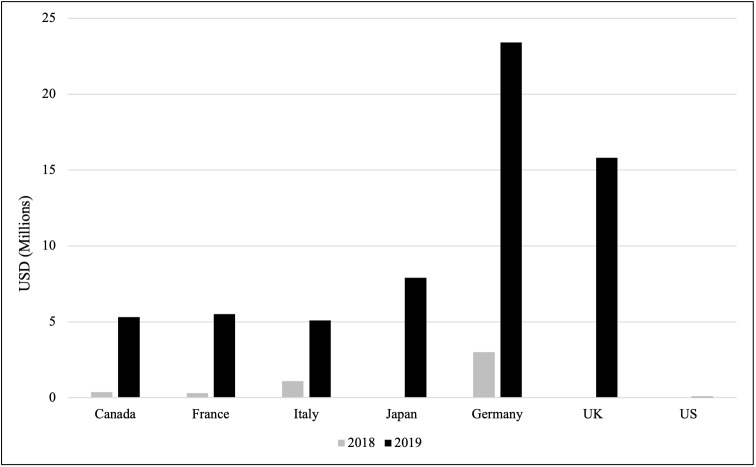
Allocation of funding to NCDs by G7 donor countries (2018–2019). A comparison of DAH allocated to NCDs by each G7 country in the years 2018 and 2019.

We also compared the DAH allocated to NCDs among the G7 donor countries during the two years that the NCD category existed in the OECD database (**Figure 10**). All seven countries allocated less than USD 5 to NCDs in 2018, with Japan, the UK, and the US all allocating USD 0. However, in 2019, every G7 country except the US allocated more than USD 5 million to NCDs, with the US allocating only USD 51 000.

## DISCUSSION

A recurrent theme in the existing global health financing literature is the discrepancy between disease burden and DAH allocation, which has become increasingly evident in the context of NCDs. Over the last 25 years, there has been a shift in the composition of the disease burden in both low- and lower-middle income countries. While communicable diseases such as HIV/AIDS, malaria, and tuberculosis remain, their prevalence has steadily decreased – in contrast to trends observed for NCDs such as hypertension, diabetes. and cancer. This shift is referred to as epidemiologic transition [9].

In sub-Saharan Africa, for instance, traumatic injury is more prevalent than tuberculosis, HIV/AIDS and malaria combined, yet ‘HIV/AIDS dominates in terms of funding, with 54% of US expenditure on global health programs being dedicated to HIV/AIDS alone’ [10]. Scheiner et al. [10] also reported that ‘although surgical disease accounts for nearly one-third of the global disease burden – with trauma serving as a substantial affliction – only 11.5% of global health spending was directed toward surgery, whereas 60.8% was directed toward infectious diseases.’ An analysis of US global surgery contributions found that the National Institute of Health (from 1991 to 2014) and the United States Agency for International Development (from 2006 to 2013) each allocated less than 1% of their total budgets to surgical disease-focussed work [11].

NCDs are often chronic and can lead to significant morbidity and mortality. Relatedly, 80% of deaths and 90% of early preventable deaths due to NCDs occur in low-income and middle-income countries. Furthermore, NCDs are affecting people in LMICs at a younger age than in high-income countries (HICs), increasing the economic impact on the individual, the family, and the country due to lost years of productive work. Young people in LMICs are also more likely to need to leave their jobs to care for older family members with NCDs. The ensuing economic toll of NCDs in LMICs is projected to reach USD 21 trillion by 2030 [12].

Considering the high and increasing human and economic cost of NCDs, one would assume a proportional allocation of DAH was being made to address NCDs, but prior analyses have not found this to be the case [13]. In 2014, Dieleman et al. [14] found that ‘relative to the size of the global burden, very little DAH is targeted toward noncommunicable diseases. The burden associated with noncommunicable diseases accounts for 49.8 percent of the all-cause burden, but DAH for noncommunicable diseases makes up only 1.5 percent of all DAH.’

In our analysis, we found that the total annual US-based funding increased significantly from 1995 to 2019. In the 1990s, funding was more evenly divided between health systems strengthening and disease-specific interventions. From 2000 to 2007, there was a dramatic increase in funding directed to disease-specific interventions. Since then, funding directed to health systems strengthening has comprised less than 13% of total US funding. The US has therefore failed to shift its funding allocation in response to WHO’s recommendations, the United Nations’ new goals, as well as the Lancet Commission’s Global Surgery: 2030 Report, which emphasises the need to invest in health systems in order to enable them to provide safe and quality care for both communicable and non-communicable diseases, including surgical disease.

Based on data obtained from the IHME Global Burden of Disease 2019 report, the percentage of deaths in LMICs attributable to NCDs has exceeded that attributable to communicable diseases since at least 1990, and this percentage has increased from 61% in 1990 to 79% in 2019. In low-income countries, which experienced the epidemiologic transition later than LMICs, communicable diseases caused a greater percentage of deaths than NCDs until 2008. Since then, the percentage of deaths attributable to NCDs in these countries has risen from 51% in 2009 to 62% in 2019. However, according to the OECD database, total donor funding for NCDs represented only 0.91% of DAH in 2019.

It is clear that the HIV/AIDS epidemic was a key driver of US funding allocation patterns over the last two decades. Initially, HIV/AIDS funding was largely directed towards prevention and health systems strengthening; yet over the next 15 years (2000–2015), the focus shifted to treatment, which is largely funded by PEPFAR due to the congressional mandate that 50% of PEPFAR funding be allocated to providing HIV/AIDS patients with antiretroviral treatment [15]. The combined percentage of total global DAH directed to HIV/AIDS and infectious disease control rose from 12.7% in 1992 to 49.1% in 2005, with the US as the highest contributor and the Bill and Melinda Gates Foundation and Gavi as significant contributors [16]. This represented a 12-fold increase in funding for HIV/AIDS from 1992 to 2005. ‘This shift in relative shares toward vertically oriented funding is one of the most noticeable of all trends in donor aid for health and population… by 2006, funding for HIV/AIDS had reached approximately 80% of US aid for health and population… Meanwhile, US funding for other health and population issues stagnated in absolute terms’ [16].

For recipient countries with historically low levels of spending on health, this rapid and exponential increase in funding posed significant challenges regarding effective implementation. In countries such as Uganda and Ethiopia, funding directed to HIV/AIDS in 2003 and 2004 exceeded the governments’ annual budgets for the entire health sector. Of note, the HIV prevalence rate in Ethiopia at that time was 1.4% [4]. In Rwanda, where the HIV prevalence rate was 3%, USD 47 million were disbursed for HIV/AIDS, compared to only USD 18 million for malaria, which was the leading cause of morbidity and mortality at that time. ‘Commenting on this issue in Rwanda, a government report noted ‘a gross misallocation of resources’ in the health sector, stating that ‘the main problem is the development partners’’ [16].

Shiffman et al. [16] raised the question of whether the substantial funds allocated to HIV/AIDS since the early 2000s can be considered as additional resources or as funds redirected from other parts of the health sector. In our current analysis, it appears that these funds might indeed be additional, as the rise in HIV/AIDS-directed funds coincides with the overall increase in total DAH from the US. However, similar to Shiffman and colleagues’ findings, we observed a concurrent decrease in the proportions of US DAH allocated to health systems strengthening and reproductive health. Whether the augmented HIV/AIDS-directed funds directly led to a reduction in funds elsewhere or not, the predominant focus on this specific intervention seems to have resulted in a displacement of aid from health systems strengthening initiatives.

Prior studies on the impact of aid allocation on health outcomes have yielded mixed results. Proposed explanations include the fungibility of DAH, meaning that increased aid provision does not necessarily equate to increased total health spending, as well as the extensive confounding factors impacting health outcomes across widely varying contexts. However, many micro-level analyses have shown significant associations between the provision of DAH targeted to a specific health area and improved health outcomes in that area. For example, Hsaio et al. [17] examined DAH targeting and health outcomes with regard to HIV, TB, and malaria in 120 LMICs and found that mortality for all three diseases declined from 2005 to 2010, the period during which total DAH increased most significantly. Furthermore, they showed that malaria DAH and HIV DAH were predictive of a decrease in malaria mortality and HIV mortality on a country level.

Today, HIC-based aid to LMICs continues to focus largely on infectious diseases despite the far greater incidence and mortality burden of NCDs. Scheiner et al. [10] offered possible explanations for this trend, one being that communicable diseases pose a threat to HICs. This may motivate HICs to prioritise both the containment of such diseases and the development of effective treatments in a less costly setting. For example, between 2014 and 2016, over USD 3.61 billion in foreign aid was allocated to combating the Ebola crisis; however, only 11 316 people died due to Ebola compared to 1.4 million people who die annually due to untreated surgical disease [10]. As reported by KFF, the United States alone allocated USD 5.4 billion towards the Ebola response in 2015, earmarking USD 909 million specifically for global health security. Subsequently, the following year witnessed the allocation of USD 1.1 billion in emergency funding from the US to address the Zika outbreak, with USD 145 million designated for global health security. These financial commitments underscore the significant investments made by the US in addressing global health crises while overlooking the fatalities resulting from untreated surgical diseases [18].

In the academic realm, health systems research has predominantly concentrated on NCDs due to their significant implications for day-to-day health system capacities. Conversely, health security research has primarily emphasised communicable diseases, given their potential for rapid transmission. Nevertheless, it is crucial to underscore that a health system equipped with processes to address NCDs is poised to function more effectively as the first line of defence in the event of a communicable disease outbreak.

In summary, the observed trends in US funding as reported in the OECD highlight a preference for supporting disease-specific interventions largely targeting communicable diseases over NCDs and health systems strengthening. While the focus on communicable diseases may have been closely aligned with global health security concerns related to epidemics spreading to HICs, the shifting global prevalence towards NCDs diminishes the impact of the US in the global health scenario, despite it being the biggest donor. Furthermore, in neglecting health systems strengthening in its aid allocation, the US has failed to address the central health aim of the SDGs set by the United Nations in 2015.

The trends in the OECD showcase that both the increasing US funds over the years that were allocated to communicable diseases and the percentage of funds displaced towards communicable diseases and away from other causes such as NCDs and health systems strengthening has created a vulnerability in the US global health security strategy. We argue that the growing prevalence of NCDs was ignored as a potential risk to global economic devastation. Furthermore, the importance of health systems strengthening was overlooked in preventing such economic damage and as an integral aspect of pandemic preparedness. To address these gaps, a realignment of US fund allocation is essential, considering the SDGs, evolving global disease patterns, and the priorities of recipient countries.

Domestic policy decisions, driven by political agendas and budget constraints, directly influence the allocation of resources to global health initiatives. International relations play a crucial role, as diplomatic ties and geopolitical strategies can impact the focus and distribution of health assistance. Additionally, economic interests, such as trade relationships and investment opportunities, often determine the prioritisation of specific health programmes and regions. By examining these interconnected factors, researchers can uncover the complex motivations behind funding decisions, identify gaps in current approaches, and propose more effective strategies for aligning DAH with the evolving needs of LMICs. This comprehensive analysis can ultimately lead to more equitable and sustainable health outcomes globally.

### Limitations

Our study had multiple limitations, largely related to the lack of available data pertaining to surgical disease and NCDs in general. The OECD database did not include a category for NCD-directed funding prior to 2018. This likely reflects the absence or negligible size of funds directed to NCDs prior to then, however this cannot be concluded with certainty. Furthermore, the OECD and most other financial global health databases continue to lack a surgical disease category to track funding allocations to this area of the health sector.

While an appropriate comparison could be made between the included time points between 1995 and 2019, it is important to note that the study aims excluded data points after the beginning of the COVID-19 pandemic. This was intentionally done to assess the funding allocation to health systems strengthening and NCDs without having to consider resource reallocation amidst a global pandemic; however, future studies may choose to reassess NCD funding allocation trends after the immediate post-COVID-19 pandemic period in the upcoming decade.

Inherent limitations must also be considered with regard to the data obtained from the OECD and IHME databases. The OECD database representation may be skewed towards its member countries and several select non-members, meaning that trends in other regions may be overlooked. Quality and accuracy of data are also impacted by differences in reporting standards and methodologies across countries. Other constraints include limited granularity in aggregation levels and availability of variables, and changes in methodologies over time. The IHME database is constrained by its reliance on modelling and estimation techniques, which may introduce uncertainties and inaccuracies, particularly in regions with sparse data or unreliable reporting. Coverage bias is another concern, as the database may disproportionately represent regions with better health care infrastructure or data collection capabilities. Despite these limitations, both databases represent invaluable sources for global health research and policy development.

## CONCLUSIONS

The global burden of disease in LMICs has and will continue to shift such that NCDs, including trauma, cancer and other surgical diseases, are the dominant cause of morbidity and mortality. The historical approach of funding primarily disease-specific interventions targeting communicable diseases has done little to strengthen the wider health systems in ways that capacitate them to address the changing disease burden of the populations they serve.

While the establishment of the MDGs and the launch of new global health initiatives resulted in a substantial increase in global health funding, it also resulted in the provision of earmarked funds to disease-specific interventions prioritised by donor countries and institutions, as opposed to unspecified funds that allow a recipient country’s health ministry to allocate funding based on their needs and priorities.

Our findings showed that, despite being the largest single contributor to DAH, the US government has not adapted its funding patterns in response to changing disease prevalence. To bridge the gaps created by misallocated funds and better address the needs of LMICs, it is crucial for the US to realign its fund allocation strategy – which should be guided by international development goals and adapted to changing disease prevalence, and which should prioritise specific needs of recipient countries (**Box 1**). In doing so, the US can play a pivotal role in advancing global health equity and resilience.

Box 1 **Recommendations for policymakers**

1. Increase funding directed to health system strengthening, including surgical capacity building, to create durable improvements in global health.
2. Increase funding for non-communicable diseases as their morbidity and mortality burden now exceeds that of communicable diseases in countries of all income classes.
3. Increase the proportion of funding that is not directed to a specific area of the health sector. This will allow recipient countries to set their own priorities based on their needs.
